# Using Behavioral Science to Design a Peer Comparison Intervention for Postabortion Family Planning in Nepal

**DOI:** 10.3389/fpubh.2016.00123

**Published:** 2016-06-21

**Authors:** Hannah Spring, Saugato Datta, Sabitri Sapkota

**Affiliations:** ^1^ideas42, New York, NY, USA; ^2^Marie Stopes International, London, UK

**Keywords:** behavioral economics, behavioral science, social comparison, postabortion family planning, family planning, sexual and reproductive health, peer comparison, behavioral design

## Abstract

Despite the provision of free and subsidized family planning services and clients’ demonstrated intentions to delay pregnancies, family planning uptake among women who receive abortion and postabortion services at Sunaulo Parivar Nepal (SPN), one of Nepal’s largest non-governmental sexual and reproductive health (SRH) providers, remains low. Through meetings, interviews, and observations with SPN’s stakeholders, service providers, and clients at its 36 SRH centers, we developed hypotheses about client- and provider-side barriers that may inhibit postabortion family planning (PAFP) uptake. On the provider side, we found that the lack of benchmarks (such as the performance of other facilities) against which providers could compare their own performance and the lack of feedback on the performance were important barriers to PAFP uptake. We designed several variants of three interventions to address these barriers. Through conversations with team members at SPN’s centralized support office and service providers at SPN centers, we prioritized a peer-comparison tool that allows providers at one center to compare their performance with that of other similar centers. We used feedback from the community of providers on the tools’ usability and features to select a variant of the tool that also leverages and reinforces providers’ strong intrinsic motivation to provide quality PAFP services. In this paper, we detail the process of identifying barriers and creating an intervention to overcome those barriers. The intervention’s effectiveness will be tested with a center-level, stepped-wedge randomized control trial in which SPN’s 36 centers will be randomly assigned to receive the intervention at 1-month intervals over a 6-month period. Existing medical record data will be used to monitor family planning uptake.

## Introduction

On average, approximately 210 million women become pregnant each year. As of 2003, fifth of these women (42 million) induce abortion, of which approximately 21 million were unsafe (1). A primary cause of these abortions is the unmet need for family planning, as evidenced by the reduced rate of abortion in countries following the introduction of national contraceptive programs (2).

A woman’s fertility can return 1 week after an abortion, highlighting the importance of postabortion family planning (PAFP) uptake (3). Research suggests that timely access to PAFP services can prevent subsequent unintended pregnancies. Consequently, practitioners aim to increase patients’ timely access to contraception by integrating counseling on family planning into postabortion services. In low-income countries, rates of contraceptive use have increased, and rates of unplanned pregnancies and repeat abortions have decreased, when women received counseling and services during the abortion process (4).

### Family Planning and Safe Abortion Service Provision in Nepal

Beginning in the late 1950s, non-governmental organizations in Nepal supported family planning programs by increasing the availability of family planning services and promoting norms around family size and the use of family planning (5). In 2007, the government of Nepal put forth the goal of providing contraceptive supplies free of charge at public health facilities. As of 2011, modern contraceptive prevalence among married and unmarried women in Nepal was 33.2%, with the most common method being sterilization (11.9% for women and 6% for men), followed by injectables (7%), condoms (3.3%), the pill (3.2%), the intrauterine device (1%), and implants (0.9%) (6).

Abortion has been legal in Nepal since 2002, and contraceptive counseling is a mandated and fundamental component of comprehensive abortion care (CAC) services (7). An evaluation of PAFP uptake across sexual and reproductive health (SRH) centers in Nepal found that approximately 250,000 women received abortions from 2009 to 2011. Furthermore, research on PAFP in Nepal suggests that only 5% of women wanted a child within 2 years of having an abortion, suggesting that a large fraction of abortion clients are likely to have a need for contraception (8). This intuition is confirmed by results from a meta-analysis of 10 studies showing that more than half of postabortion care (PAC) clients expressed an interest in using contraception (9). Six of the studies included in this meta-analysis also show that only about one-quarter (27%) of postabortion clients left the SRH facility where they received an abortion with a contraceptive method (9). Taken together, this evidence suggest that Nepali women receiving abortions have a desire to limit or space births using contraception, but most are not currently able to fulfill this desire. Other studies have found PAFP uptake rates as high as 80 to 98% in low-income countries like Tanzania, Zimbabwe, and Malawi, following the integration of PAFP services with abortion service provision (10). Thus, there is both a need for and scope to design and test innovative interventions to increase PAFP in Nepal.

### PAFP Uptake at Sunaulo Parivar Nepal’s 36 Sexual and Reproductive Health Centers

Sunaulo Parivar Nepal (SPN), an implementing partner of Marie Stopes International, approached us to work on improving PAFP uptake in their 36 centers. SPN is one of the largest providers of SRH services in Nepal, providing an estimated 55% of sterilizations and 89% of safe abortion services in 2010 and 2011. We entered into a partnership with SPN to collaboratively understand barriers to PAFP uptake and design interventions to improve PAFP uptake.

Sunaulo Parivar Nepal defines PAFP uptake as a woman’s decision and action to use a modern contraceptive method within 2 weeks of receiving a safe abortion or postabortion service. Service providers from SPN’s 36 SRH centers oversaw 130,000 client visits in 2014 and provided approximately 40,000 medical and surgical abortion services, including PAC services, in 2014. SPN’s centers are well-suited for the design of behaviorally focused interventions to increase PAFP uptake for several reasons. First, current uptake rates are relatively low, with average PAFP uptake rates between January 2015 and June 2015 ranging from 38 to 44%. Second, SPN has made considerable investments in counseling training. Finally, SPN’s 36 centers have ready and adequate supplies of contraception (including injectables, implants, IUDs, pills, condoms, etc.), suggesting that the primary bottlenecks restricting PAFP uptake are likely behavioral rather than driven by access or infrastructure.

### Suitability and Effectiveness of Behavioral Economics Approach

In SPN’s centers, there are a series of decisions and actions, both conscious and unconscious, that both providers and clients must make. These decisions and actions determine PAFP uptake, including decisions around which specific method to use. Behavioral economics provides a useful framework to analyze such decision-making, given that it often reveals influences on decisions and actions, which are alterable. For example, behavioral economics has proven useful in bridging the gap between intentions and actions where these diverge. Specifically, in cases where people make a choice or have a desire for a certain outcome, interventions informed by behavioral economics help them follow through with the actions to achieve that outcome. In the case at hand, behavioral economics can provide effective tools to help identify barriers that may be standing in the way of strongly motivated, well-trained providers’ ability to administer quality services to clients, despite their desire to do so.

## Materials and Methods

In the following section, we explain the participatory processes used to understand and design for the underlying causes of low family planning uptake postabortion at SPN’s 36 SRH centers. First, we explain how a behavioral economics method was used throughout the process. Later, we describe how we conducted qualitative research to understand the context, perceptions and behaviors, and the iterative design and user testing process we used to create and refine our interventions. This paper focuses on provider-related contextual features, behaviors, and perceptions that could be inhibiting PAFP uptake.

A description of all the qualitative research conducted during this partnership is in Table 1. The findings from the qualitative research and design processes are presented in the Section “Results.”^1^

**Table 1:** **Summary of qualitative research**.^a^

Purpose of qualitative research	Description of qualitative research	Date of qualitative research
Narrow down potential scope of problems to address through this partnership	13 client interviews and 6 provider interviews	August 2014
Explore behavioral and contextual drivers of barriers to PAFP uptake	31 client interviews and 8 provider interviews	December 2014
Explore themes related to provider motivation and role sharing	Phone interviews with four providers	August 2015
Receive feedback on design concepts	User testing and conversations with eight providers	November 2015

*^a^Note, given natural disasters, resource shortages, and political instability in Nepal, the qualitative research process occurred over a longer period of time than originally planned*.

### Behavioral Economics Approach

We used a behavioral economics approach to inform the three stages of this research: prioritizing one specific behavioral challenge to focus on, determining the underlying features of the context that contribute to that challenge, and designing behaviorally informed solutions to overcome the challenge and improve outcomes. In order to decide which problem to focus on, we compared PAFP uptake rates at SPN’s centers to uptake rates at other comparable health centers. We also considered various aspects of the abortion and postabortion process and compared uptake rates for specific parts of that process. Next, we undertook the behavioral diagnosis process, charting out the decisions and actions of individuals (clients and providers) and using theories from behavioral science to identify why people might be failing to act on their intentions. This process allows us to generate hypotheses about what might be influencing the behavior. Finally, we designed to the contextual feature causing the identified challenge. In this case, each stage is informing the subsequent stage.

### Qualitative Research: Narrowing the Scope of Potential Areas to Intervene

We conducted initial qualitative research at nine SRH centers in urban and rural Nepal over two-week-long site visits in 2014. This research included direct observation of the counseling and intake processes at the health centers, as well as a total of 44 interviews with clients and 14 interviews with family planning service providers and counselors.

We conducted observations of client check-in, pre-abortion, and family planning counseling, the time between counseling and abortion provision, and postabortion recovery procedures.

We conducted interviews with clients during various stages of the abortion and family planning uptake processes, including pre-counseling, post-counseling, pre-abortion, and postabortion. Women were asked about their prior use of contraception and familiarity with different methods, along with their intention to take up contraception during this process. Women were also asked to give more general feedback on their experiences at the center and with the providers.

We conducted interviews with family planning service providers and counselors. We asked about their history of working with SPN, their typical day, the services they provide, their clients, and their attitudes and motivations around service provision.

Each stage of qualitative interviews informed the later. After each round of interviews, our focus and purpose became narrower. By the final round of interviews, we focused in on barriers to providers’ perceptions of their center’s performance and the performance of other centers.

The research team at SPN conducted further interviews with four providers in August 2015. These interviews focused on the providers’ prioritization of responsibilities, their positive motivation for provision of abortion and family planning services, and their attitudes in the workplace. During the interviews, the research team asked providers to explain what performing “well” meant to them and how they thought their performance compared to that of other providers. The research team also asked counselors and providers to give feedback on the counseling process and the current service provision benchmarks, in addition to talking more generally about their own strategies for managing their workloads. Findings from these interviews are presented in the Section “Results.”

### Design Generation and User Testing: Behaviorally Informed Interventions to Address Barriers

The qualitative research described above gave us a better understanding of service providers’ and counselors’ decisions and actions. Given findings related to the variance in quality of counseling provided, we decided to focus on barriers to optimal provider behavior. Specifically, we focused on improving the consistency with which providers counseled clients on family planning methods.

We worked with SPN to identify and prioritize intervention designs. Three interventions were user-tested with a total of eight service providers, team leaders, and counselors from five centers in Kathmandu. Providers played a key role in choosing which intervention would be tested in the randomized controlled trial.

During the user testing sessions, we observed center staff as they interacted with three variants of each of the three interventions. We asked about their first impressions, and the meaning they drew from the interventions. Next, we asked them to provide candid feedback on the elements of each, including document titles, the centers in the comparison group, and the use of different symbols to indicate a center’s progress compared to other SRH centers.

The outcomes of these sessions are presented in the Section “Results.” For the purpose of this paper, we will only discuss details of these user testing sessions for the three variations of the intervention chosen by the center staff.

## Results

In this section, we describe the insights gleaned through qualitative research that relate to provider behavior and perceptions. Next, we explain how we worked with providers to design interventions that increase PAFP uptake.

### Results from Qualitative Research with Providers

Results from observations and interviews with providers showed that they were intrinsically motivated to offer high-quality services and knowledgeable on best practices related to family planning service provision and counseling. When asked why they chose to work with SPN at an SRH center, service providers expressed compassion for their clients. Providers stated that they feel like clients are their family, and that they appreciate the opportunity to help women in need. Providers also remarked that they become emotional when they hear their clients’ stories, and when their clients are in bad situations.

Service providers were knowledgeable on the most effective ways to counsel women. Interviewed providers described the correct protocol for using the balanced counseling method and had adopted personalized ways of explaining certain clinical or physiological aspects of family planning and side effects to women.

While these interviews suggested that SRH providers were intrinsically motivated to offer high-quality services and were well-trained and informed about the modalities of counseling clients on contraceptive options, they nevertheless faced significant barriers to consistently providing family planning counseling and service. The key insight from our research was that despite providers being well-trained and motivated, the existing systems made it hard for them to clearly place their own performance into a broader context, and thus made it difficult for them to assess how successfully they were carrying out their duties. In particular, providers lacked information about how center-level family planning uptake in their center compared with uptake in other centers. Absent this information, it was very hard for them to assess how well they were doing, whether they could improve performance on PAFP uptake, and by how much. In addition, in SRH centers with large teams, role sharing and the diffusion of responsibility made it easier to assume that another provider might be providing family planning counseling, which could hinder consistent counseling.

The key barrier standing in the way of providers administering more consistent PAFP counseling, which in turn was stymieing efforts to increase PAFP uptake, was thus the lack of access to information on PAFP uptake rates of other centers. When asked how providers know their own center’s PAFP uptake rates, all providers referenced their daily and monthly records related to both PAFP outcomes and comprehensive abortion and PAC statistics more broadly. However, when asked how they know the performance of other centers, providers had mixed answers. One provider said they gathered this information through announcements made during quarterly meetings, while another referenced conversations with the support office or other centers. Two providers implied that most providers do not have this information: either no one within the center has information about other centers or only the team leaders have this information.

Finally, results from interviews suggested that role sharing could be hindering consistent counseling of all women. When asked what providers do when particularly busy, all of the providers interviewed stated that they share roles with other team members. Role sharing, particularly without adequate communication, could lead to diffusing responsibility between team members. This could result in inconsistent service provision for some clients, particularly on days when client flow at centers is high.

### Behavioral and Contextual Barriers to PAFP Uptake

The findings related to provider perceptions and behavior that were most relevant to understanding barriers to PAFP were the lack of information on other center’s PAFP uptake and the use of role sharing in order to manage overcrowding in centers.

The theorized mechanisms for how these perceptions and behaviors lead to lower PAFP uptake is described below.

Individuals seeking to assess their own performance or behavior often look to social cues or markers to determine whether their performance is satisfactory or in need of improvement. They may do this by directly observing others’ actions or reactions to determine what behavior is socially appropriate or acceptable (11), or by seeking out objective information that allows them to assess their own opinions and abilities (12). In the case of PAFP providers, this process of social comparison was hindered by the absence of information on the PAFP uptake rates achieved by other SPN centers. This meant that the providers were unable to accurately assess their performance against that of comparable peers. They therefore had no way of knowing whether their performance was in need of improvement, so thus had difficulty determining whether they should use other strategies to increase PAFP uptake. Such strategies might include reviewing the balanced counseling protocol, or taking measures to ensure that every woman was counseled on family planning.

In addition, role sharing likely made it more difficult to ensure that every woman was counseled on family planning services. Role sharing without ample communication can cause certain parts of the complicated abortion and PAFP processes to be neglected. Given that multiple team members are trained on counseling, due to role sharing certain clients may not be counseled or offered family planning. Through center observations, we found a clear example of this type of oversight. Women who returned to the center for their 2-week follow-up visit were not counseled on family planning, perhaps because the burden of care for these types of follow-up visits was shared among many different providers, who could not always communicate specifics of each case to the various providers and counselors. Diffusion of responsibility from ineffective role sharing could lead to inaction among team members with similar roles.

Given the lack of adequate information to make social comparisons, we suggested implementing an intervention that give providers access to information on their center’s PAFP uptake compared to PAFP uptake at other similar centers. This type of information would allow providers to gage their performance and strategize ways to improve performance. In addition, sending this information to SPN’s centers monthly, to be discussed by all team members during regular team meetings, would facilitate the type of communication necessary for effective role sharing and for preventing diffusion of responsibility. Finally, through prompts on the intervention, providers will be encouraged to discuss best practices for counseling, as well as techniques to ensure every woman receives counseling.

In addition to the mechanisms already mentioned, the intervention also leverages providers’ existing intrinsic motivation. The intervention will provide recognition to high-performing centers and emphasize their exemplary care for patients. Similar peer-comparison interventions have been shown to work well for individuals who have strong intrinsic motivation and have been successfully used to improve outcomes related to condom sales, energy use, and outdoor water consumption (13–15).

We hypothesize that monthly posters comparing centers’ PAFP uptake rates will change provider behavior by giving them the necessary information to put their performance in the context of other centers’ performance. By highlighting room for improvement across centers, this information will lead providers to strategize ways to improve PAFP uptake performance. We hypothesize that the resulting improved quality and frequency of counseling will increase the likelihood that safe abortion and postabortion clients will take up family planning.

### Results from Behaviorally Informed Design Generation and User Testing

During the user testing sessions, center staff interacted with three potential interventions, demonstrating preference for the monthly center-based PAFP comparison poster. We designed three variations of this poster. To maximize fidelity of the designs, we reviewed findings from evaluations of peer-comparison interventions from other settings (16). Based on this, we crafted reference groups of centers such that they would have comparable volumes of patient flow, numbers of center staff, and PAFP uptake. In addition, we added encouraging language and loss aversion language to avoid the boomerang effect.

The principal feature, present in all three variations, was numerical information on the center’s PAFP uptake compared to that of other similar centers. Version One compared the center’s PAFP uptake to several other centers whose names were listed. The comparison was made using a bar graph and a text box cuing positive or negative reinforcement. Version Two compared the center’s performance to the average PAFP uptake of top-performing centers and of comparable centers. Like Version One, Version Two showed the uptake comparison visually with a bar graph and through a text box with positive or negative reinforcement. Version Three sent centers a monthly “model center” poster describing the highest performing center, the services rendered at the “model center,” and the PAFP uptake of the center receiving the poster. This version allowed the center to set a goal for PAFP uptake and read about the monthly “model center’s” strategies for increasing uptake. Smaller variations included adding different symbols, images, and cues to promote a positive or negative affect. These ranged from smiley faces, stars, and trophies to green or red color schemes.

Results from user testing with providers showed that Version One was the most compelling, easy-to-understand, and desirable version of the poster. Providers looked at this document and quickly knew what their centers’ PAFP uptake rates were and how they compared to other centers. Providers felt that the other centers provided a fair comparison. This intervention also created increased social accountability among providers, more so than the other versions of the intervention, which did not compare uptake among centers.

## Discussion

A participatory approach to identifying barriers and designing solutions has proven productive in the current context. This is likely due in part to the strong stated desire of providers and counselors to improve outcomes. In addition, service providers and counselors had a preference for certain interventions and were willing to openly share this preference.

The novelty of this intervention is the process that was taken to determine which intervention to use, which was highly participatory and allowed us to respond to the barriers present in this specific context. Benchmarking interventions have proven effective in other health, retail, and workplace settings, in both developed and developing countries (15, 17, 18). However, best practices for the implementation and evaluation of these interventions are still being developed.

While the researchers believe that this intervention should improve outcomes related to PAFP uptake, especially given the iterative and participatory research and design processes, the effectiveness of the intervention will be evaluated rigorously. The impact evaluation will use a clustered, stepped-wedge, randomized controlled design. The intervention will be implemented and evaluated in SPN’s 36 SRH centers spread across the 31 districts and 5 regions of Nepal. Providers from all 36 centers, and women of reproductive age seeking safe abortion services at those centers, will be eligible to participate in the study. Assuming the test is run for 6 months, and a minimum detectable effect of 4.0%, the sample size will be approximately 11,500 safe abortion clients.

All centers will begin in the control group. Centers will be randomly assigned to the treatment group in sets of nine centers. Each month, additional nine centers will be added to the treatment group and will begin receiving the intervention. The outcome measure, family planning uptake, will be collected through existing medical records. The data will be electronically shared between SPN centers and SPN’s centralized support office and will be shared with our team monthly.

After completion of the randomized controlled trial, SPN will decide whether or not to implement this intervention in its 36 SRH centers.

## Conclusion

A participatory approach to research and intervention design is beneficial for determining which barriers to address through an intervention and which specific features of an intervention to include. This paper details the processes and results from a participatory approach to research and intervention development with SPN in Nepal. We identified several actionable barriers to the provision of PAFP and designed a low-cost, easy to implement solution to overcome these barriers with the feedback from service providers and counselors who have a desire to improve outcomes at their SRH centers.

Summary of provider qualitative interview guides:

- Personal history of working with SPN
- Description of typical day at work
- Community perception of SPN, comprehensive abortion care, and use of family planning
- Description of key services provided, including processes for providing those services
- Description of types of women served
- Explanation of current knowledge, attitudes and preferences of women served
- Opinions on why women may or may not decide to take up family planning
- Role play counseling process, follow up visit
- Perceptions of current PAFP uptake levels, including how their levels compare to other centers, and any barriers they may face to increasing PAFP uptake
- Description of team member roles, coordination, and communication

Summary of client qualitative interview guides:

- Description of typical day
- Community perception of SPN, comprehensive abortion care, and use of family planning
- Perception of services at SPN
- Knowledge and preferences related to family planning methods
- Preferences and priorities related to various attributes of family planning methods

Summary of user-testing guides:

- Initial thoughts after reading the tool
- Initial feeling or emotions after reading the tool
- Feedback on what stood out visually about the tool
- Feedback on various visual and text aspects of the tool
- Feedback on how other centers may respond to the tool
- Feedback on anything that should be added or removed

## Author Contributions

The author HS did the majority of the research and intervention design described in this paper and also completed a large portion of the writing for this paper. The author SD oversaw the behavioral research and intervention design described in this paper and also did the majority of editing for the paper. The author SS oversaw research-related aspects of this project from the Marie Stopes Nepal and Marie Stopes International teams and also provided edits to many versions of the paper. The author SS provided representation from Marie Stopes International and Marie Stopes Nepal.

## Conflict of Interest Statement

The authors declare that the research was conducted in the absence of any commercial or financial relationships that could be construed as a potential conflict of interest. The reviewer (JH) and the handling Editor declared a current collaboration, and the handling Editor states that the process nevertheless met the standards of a fair and objective review.

## Footnotes

^1^We obtained written consent from both providers and clients before conducting the research described below.
